# MiR‐485‐3p modulates neural stem cell differentiation and proliferation via regulating TRIP6 expression

**DOI:** 10.1111/jcmm.14743

**Published:** 2019-11-15

**Authors:** Juxian Gu, Rusheng Shao, Meng Li, Qiuyue Yan, Hongwei Hu

**Affiliations:** ^1^ Department of Neurology Cangzhou Central Hospital Cangzhou China; ^2^ Department of Pain Cangzhou Central Hospital Cangzhou China

**Keywords:** Alzheimer's disease, miR‐485‐3p, neural stem cells, TRIP6

## Abstract

Recent references have showed crucial roles of several miRNAs in neural stem cell differentiation and proliferation. However, the expression and role of miR‐485‐3p remains unknown. In our reference, we indicated that miR‐485‐3p expression was down‐regulated during NSCs differentiation to neural and astrocytes cell. In addition, the TRIP6 expression was up‐regulated during NSCs differentiation to neural and astrocytes cell. We carried out the dual‐luciferase reporter and found that overexpression of miR‐485‐3p decreased the luciferase activity of pmirGLO‐TRIP6‐wt but not the pmirGLO‐TRIP6‐mut. Ectopic expression of miR‐485‐3p decreased the expression of TRIP6 in NSC. Ectopic miR‐485‐3p expression suppressed the cell growth of NSCs and inhibited nestin expression of NSCs. Moreover, elevated expression of miR‐485‐3p decreased the ki‐67 and cyclin D1 expression in NSCs. Furthermore, we indicated that miR‐485‐3p reduced proliferation and induced differentiation of NSCs via targeting TRIP6 expression. These data suggested that a crucial role of miR‐485‐3p in self‐proliferation and differentiation of NSCs. Thus, altering miR‐485‐3p and TRIP6 modulation may be one promising therapy for treating with neurodegenerative and neurogenesis diseases.

## 1 INTRODUCTION

Neural stem cells (NSCs) in mammalian brain possess two necessary properties of the stem cells, multipotency and self‐renewal, and it has ability to differentiate to new neuron that can function into extant neural circuits.^1^, ^2^, ^3^, ^4^, ^5^ The transplantation of NSCs supplies a potential therapeutic way for several neurological disorders including Parkinson's disease, Alzheimer's disease, Huntington's disease and spinal cord injuries.^6^, ^7^, ^8^, ^9^, ^10^, ^11^ Despite the great accomplishment has been achieved, there are still some challenges to resolve before clinical use of NSCs was adopted.^12^, ^13^, ^14^, ^15^ Therefore, it is crucial to exploit the molecular signal pathway and molecular mechanism modulating NSCs differentiation and proliferation.

MicroRNAs (miRNAs) are a class of noncoding, short RNAs molecules that negatively modulate gene expression through binding a perfectly complementary or a partially complementary sequence in 3′‐untranslated region (UTR) region of their target gene to influence mRNA stability and/or translation.^16^, ^17^, ^18^, ^19^ Altered specific miRNAs expression has been shown in diverse human tumours such as breast cancer, lung cancer, prostate cancer, osteosarcoma and hepatocellular carcinoma.^20^, ^21^, ^22^, ^23^, ^24^, ^25^ Increasing studies also demonstrated that miRNAs involved in the process of diverse cell biological processes including cell differentiation, growth, migration, metastasis and invasion.^26^, ^27^ More recently, growing evidence suggested that miRNAs play important roles in the differentiation and proliferation of NSCs.

In this study, we indicated that miR‐485‐3p expression was down‐regulated during NSCs differentiation to neural and astrocytes cell. Ectopic miR‐485‐3p expression suppressed the cell growth of NSCs and inhibited nestin expression of NSCs.

## 2 MATERIALS AND METHODS

### 2.1 Cell culture and transfection

NSCs were isolated and cultured using previous standard way.^28^, ^29^ These cells were isolated from embryos of rat and kept in growth medium supplement with bFGF, EGF and N2. This reference was agreed with our hospital's ethical board and complied with Helsinki Declaration. miR‐485‐3p, miR‐485‐3p control (scramble), pcDNA‐control and pcDNA‐TRIP6 were bought from GenePharma and then transfected to NSCs by Lipofectamine with the final concentration of 10 nmol/L.

### 2.2 qRT‐PCR

RNA from NSCs was gained using TRIzol kit (Invitrogen) by standard way. qRT‐PCR was used to analyse miR‐485‐3p and mRNA expression on Applied Biosystems machine (Applied Biosystems) utilizing TaqMan mix and primer for 45 cycles. miR‐485‐3p expression was related to U6, and GAPDH was done as control for mRNA. The primers were shown: Nestin, 5′‑GATCTAAACAGGAAGGAAATCCAG G‑3′; and 5′‑TCTAGTGTCTCATGGCTCTGGTTTT‑3′; Tuj1, 5′‑CGCCATGTTCAGACGCAAG‑3′ and 5′‑CTCGGACACCAGGTCGTTCA‑3′; Ki‐67, 5′‑CAGTACTCGGAATGCAGCAA‑3′ and 5′‑CAGTCTTCAGGGGCTCTGTC‑3′; GAPDH, 5′‑ATTCCATGGCACCGTCAAGGCTGA‑3′ and 5′‑TTC TCCATGGTGGTGAAGACGCCA‑3′.

### 2.3 Cell viability

Cell growth of NSCs was detected with MTT (3‐(4,5‐dimethylthiazol‐2‐yl)‐2,5‐diphenyl‐tetrazolium bromide) assay. The OD (absorbance) at the 490 nm was recorded by microplate reader. The cell viability at 0, 1, 2 and 3 days was analysed.

### 2.4 Dual‐luciferase assay

Full‐length 3′UTR of TRIP6 gene and one fragment consisting of putative miR‐485‐3p binding site was amplified from genomic DNA and then specific cloned into pGl3‐promoter plasmid (Promega). A mutant plasmid in seed area of miR‐485‐3p binding site was also established. Cell was treated with one mixture of Renilla, miR‐485‐3p mimic, miR‐NC, pLuc‐3′‐UTR and mut or WT pGl3‐TRIP6 plasmid using Lipofectamine. Luciferase activity was detected with Promega Dual‐Luciferase kit.

### 2.5 Western blot analysis

Western blot assay was done using the standard way. Protein was isolated with SDS‐PAGE (12%) and diverted to PVDF membrane (Millipore, USA). After blocking with milk (5%) for 2 hours, membrane was stained in primary antibodies (anti‐TRIP6 and anti‐GAPDH, 1:1,000, Abcam) at 4°C overnight. After washing in TBST, membrane was incubated in second antibody. Blot was observed with ECL detection reagent.

### 2.6 Immunohistochemistry

Cell was fixed by paraformaldehyde (4%) in PBS for about 10 min and blocked with TritonX‐100 (0.1%), FBS (1%) and serum. Then, cell was stained in primary antibodies (anti‐nestin and anti‐Tuj1, 1:2,000, Abcam) at 4°C overnight. After washed three times in PBS, cell was incubated with second antibody for 1 hour at 37°C. Cell was observed by Leica camera (Leica Germany).

### 2.7 Statistical analysis

Result was present as the mean ± standard deviation and was calculated via SPSS 17.0 software. Student's t test was utilized to determine the difference between these two groups. A *P* value < .05 was regarded to be significant.

## 3 RESULTS

### 3.1 NSCs have self‐proliferation and differentiation capacity

**Figure 1:**
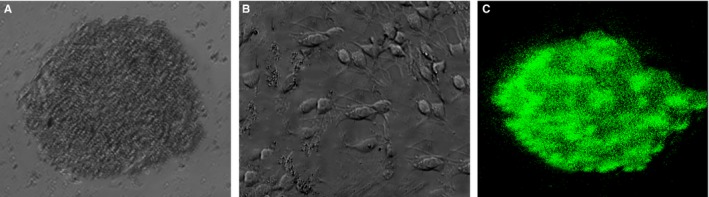
NSCs have self‐proliferation and differentiation capacity. A, NSCs were isolated from mouse forebrain and they can form neurosphere. B, These cells differentiated to astrocytes and neurons. C, NSCs were expressed the nestin, which is the NSC maker

NSCs were isolated from mouse forebrain and they can form neurosphere (Figure 1A). After withdraw of bFGF, these cells differentiated to astrocytes and neurons (Figure 1B). Moreover, NSCs were expressed the nestin, which is the NSC maker (Figure 1C).

### 3.2 miR‐485‐3p is down‐regulated a during cell differentiation of NSC

**Figure 2:**
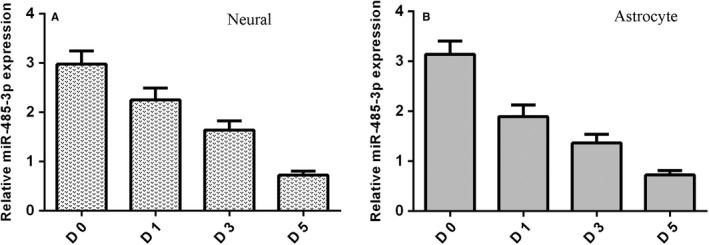
miR‐485‐3p is down‐regulated a during cell differentiation of NSC. A, miR‐485‐3p expression level was measured by qRT‐PCR assay. B, The expression of miR‐485‐3p was reduced during NSCs differentiation to astrocytes cell

miR‐485‐3p expression level was measured by qRT‐PCR assay during differentiation of NSCs. It was shown that miR‐485‐3p expression was down‐regulated during NSCs differentiation to neural cell (Figure 2A). We also found that expression of miR‐485‐3p was reduced during NSCs differentiation to astrocytes cell (Figure 2B).

### 3.3 TRIP6 is overexpressed during NSC differentiation

**Figure 3:**
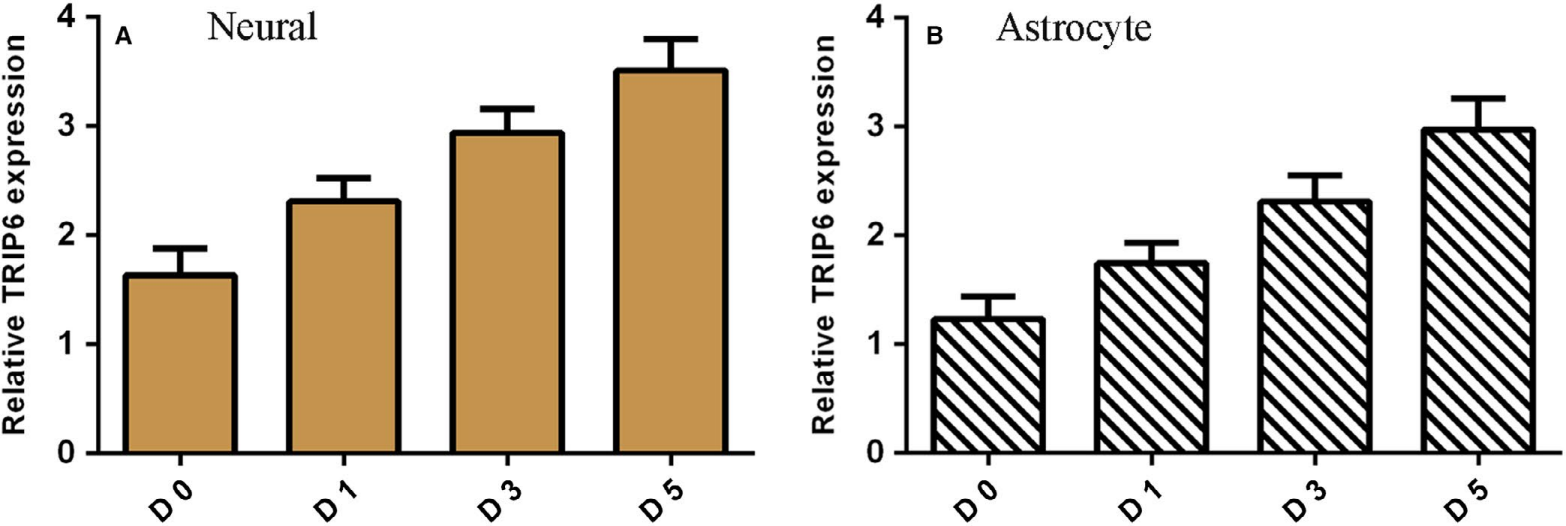
TRIP6 is overexpressed during NSC differentiation. A, TRIP6 expression level was determined by qRT‐PCR assay. B, The expression of TRIP6 was overexpressed during NSCs differentiation to astrocytes cell

TRIP6 expression level was determined by qRT‐PCR assay during differentiation of NSCs. It was shown that TRIP6 expression was up‐regulated during NSCs differentiation to neural cell (Figure 3A). We also found that expression of TRIP6 was overexpressed during NSCs differentiation to astrocytes cell (Figure 3B).

### 3.4 miR‐485‐3p targets TRIP6 expression in NSC

**Figure 4:**
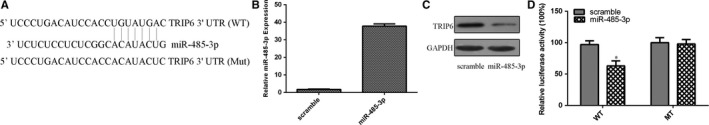
miR‐485‐3p targets TRIP6 expression in NSC. A, It was shown that miR‐485‐3p has target sites in 3′‐UTR of TRIP6. B, The expression of miR‐485‐3p was detected by qRT‐PCR assay. C, Ectopic expression of miR‐485‐3p decreased the expression of TRIP6 in NSC. D, Dual‐luciferase reporter analysis was carried out to confirm that overexpression of miR‐485‐3p decreased the luciferase activity of pmirGLO‐TRIP6‐wt but not the pmirGLO‐TRIP6‐mut. *P < .05

To find potential target gene of miR‐485‐3p, we exploited TargetScan software. It was shown that miR‐485‐3p has target sites in 3′‐UTR of TRIP6 (Figure 4A). qRT‐PCR assay demonstrated that miR‐485‐3p was up‐regulated in the NSCs after transfected with miR‐485‐3p mimic (Figure 4B). Ectopic expression of miR‐485‐3p decreased the expression of TRIP6 in NSC (Figure 4C). Dual‐luciferase reporter analysis was carried out to confirm that overexpression of miR‐485‐3p decreased the luciferase activity of pmirGLO‐TRIP6‐wt but not the pmirGLO‐TRIP6‐mut (Figure 4D).

### 3.5 miR‐485‐3p reduced proliferation and induced differentiation of NSCs

**Figure 5:**
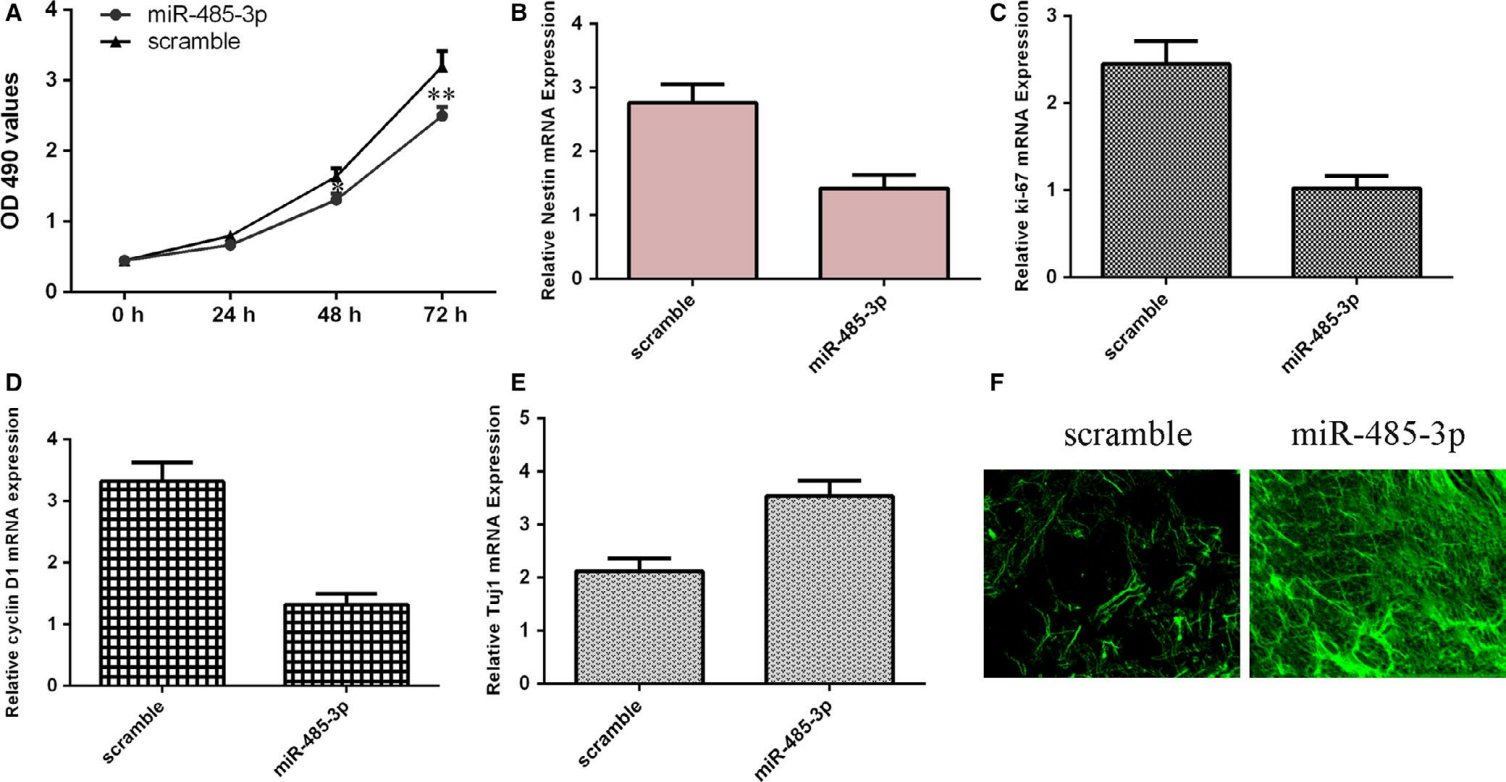
miR‐485‐3p reduced proliferation and induced differentiation of NSCs. A, Ectopic expression of miR‐485‐3p suppressed the cell growth of NSCs. B, Elevated expression of miR‐485‐3p decreased the expression of nestin. C, Ectopic expression of miR‐485‐3p suppressed the expression of ki‐67. D, miR‐485‐3p overexpression inhibited the cyclin D1 expression. E, Overexpression of miR‐485‐3p induced the Tuj1 expression, which is a maker of neuronal. F, As measured by Tuj1 immunofluorescence analysis, data showed that elevated expression of miR‐485‐3p increased the Tuj1 expression. *P < .05 and **P < .01

Ectopic expression of miR‐485‐3p suppressed the cell growth of NSCs by using MTT assay (Figure 5A). Elevated expression of miR‐485‐3p decreased the expression of nestin, which is one maker of NSCs (Figure 5B). As determined by qRT‐PCR, ectopic expression of miR‐485‐3p suppressed the expression of ki‐67 (Figure 5C). Moreover, we proved that miR‐485‐3p overexpression inhibited the cyclin D1 expression (Figure 5D). Furthermore, overexpression of miR‐485‐3p induced the Tuj1 expression, which is a maker of neuronal (Figure 5E). As measured by Tuj1 immunofluorescence analysis, data showed that elevated expression of miR‐485‐3p increased the Tuj1 expression (Figure 5F).

### 3.6 miR‐485‐3p reduced proliferation and induced differentiation of NSCs via targeting TRIP6 expression

**Figure 6:**
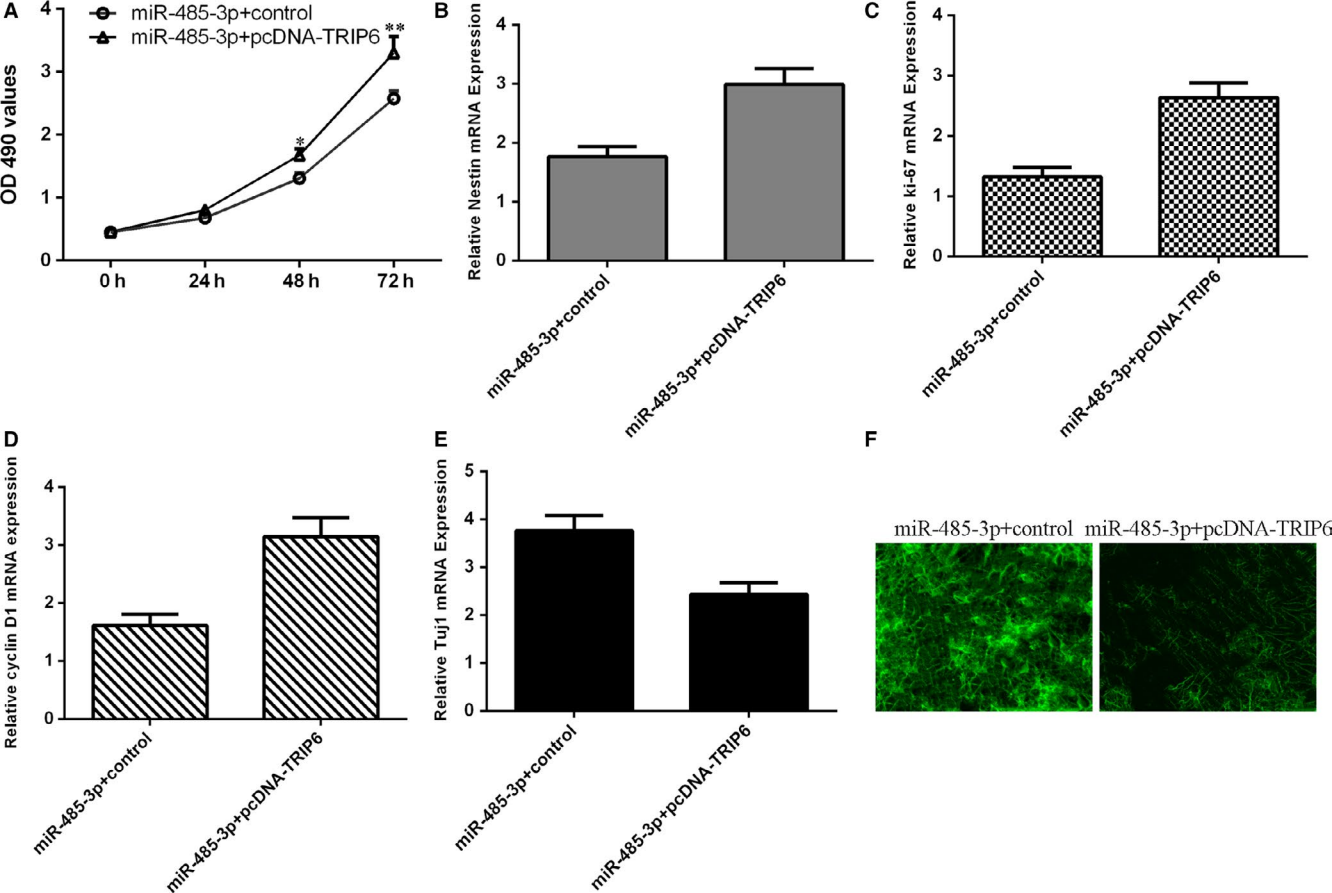
miR‐485‐3p reduced proliferation and induced differentiation of NSCs via targeting TRIP6 expression. A, Ectopic expression of TRIP6 increased miR‐485‐3p‐overexpressing NSCs proliferation with using MTT assay. B, The expression of nestin was determined by using qRT‐PCR assay. C, The expression of ki‐67 was measured by using qRT‐PCR assay. D, The expression of cyclin D1 was measured by using qRT‐PCR assay. E, Restoration expression of TRIP6 over‐turned the function effect of miR‐485‐3p on NSCs differentiation. F, As measured by Tuj1 immunofluorescence analysis, results indicated that restoration expression of TRIP6 decreased the Tuj1 expression in the miR‐485‐3p‐overexpressing NSCs. ^*^ *P* < .05 and ^**^ *P* < .01

To further consider contribution of TRIP6 to cell biological effect of miR‐485‐3p on differentiation and proliferation of NSCs, we induced TRIP6 expression in the NSCs and co‐transfected with miR‐485‐3p mimic. Ectopic expression of TRIP6 increased miR‐485‐3p‐overexpressing NSCs proliferation with using MTT assay (Figure 6A). Elevated expression of TRIP6 promoted the expression of nestin in miR‐485‐3p‐overexpressing NSCs (Figure 6B). Elevated expression of TRIP6 enhanced the expression of ki‐67 (Figure 6C) and cyclin D1 (Figure 6D) in the miR‐485‐3p‐overexpressing NSCs. Restoration expression of TRIP6 over‐turned the function effect of miR‐485‐3p on NSCs differentiation (Figure 6E). As measured by Tuj1 immunofluorescence analysis, results indicated that restoration expression of TRIP6 decreased the Tuj1 expression in the miR‐485‐3p‐overexpressing NSCs (Figure 6F).

## 4 DISCUSSION

In the present research, we found that miR‐485‐3p expression was down‐regulated during NSCs differentiation to neural and astrocytes cell. In addition, the TRIP6 expression was up‐regulated during NSCs differentiation to neural and astrocytes cell. We carried out the dual‐luciferase reporter and found that overexpression of miR‐485‐3p decreased the luciferase activity of pmirGLO‐TRIP6‐wt but not the pmirGLO‐TRIP6‐mut. Ectopic expression of miR‐485‐3p decreased the expression of TRIP6 in NSC. Ectopic miR‐485‐3p expression suppressed the cell growth of NSCs and inhibited nestin expression of NSCs. Moreover, elevated expression of miR‐485‐3p decreased the ki‐67 and cyclin D1 expression in NSCs. Furthermore, we indicated that miR‐485‐3p reduced proliferation and induced differentiation of NSCs via targeting TRIP6 expression. These data suggested that a crucial role of miR‐485‐3p in self‐proliferation and differentiation of NSCs. Thus, altering miR‐485‐3p and TRIP6 modulation may be one promising therapy for treating with neurodegenerative and neurogenesis diseases.

Increasing evidence indicated that miR‐485 has involved in the progression of varied diseases such as oesophageal cancer, glioma, osteosarcoma, hepatocellular carcinoma, osteoarthritis.^30^, ^31^, ^32^, ^33^ For instance, Chen and workmates found that miR‐485‐5p expression was negatively related with differentiation degree of bone marrow mesenchymal stem cells (BMSCs).^34^ Ectopic miR‐485‐5p expression suppressed cartilage surface‐related genes and toluidine blue, while promoted tumour necrosis factor and interleukin partly regulating SOX9 expression. It has been shown that miR‐485‐5p overexpression decreased breast tumour development and promoted chemosensitivity partly via modulating survivin expression.^35^ Previous study indicated that miR‐485‐5p expression was down‐regulated in the serum of NSCLC cells and patients. Epigallocatechin‐3‐gallate (EGCG) inhibited cancer stem cells characteristics through regulating RXRα/miR‐485‐5p axis.^36^ Du et al^37^ demonstrated that overexpression miR‐485‐3p suppressed osteosarcoma cell colony formation, growth, sphere formation and migration and inhibited CtBP1 expression. However, the role of miR‐485‐3p in NSC differentiation and proliferation remains unknown. In this reference, we showed that miR‐485‐3p expression was down‐regulated during NSCs differentiation to neural and astrocytes cell. Ectopic miR‐485‐3p expression suppressed the cell growth of NSCs and inhibited nestin expression of NSCs. Moreover, elevated expression of miR‐485‐3p decreased the ki‐67 and cyclin D1 expression in NSCs.

TRIP6 is one member of zyxin family of the LIM proteins and is one focal adhesion element with the capacity to the shuttle between cell nucleus and surface.^38^, ^39^ TRIP6 was played roles in modulation of signal transduction and actin dynamics during cell migration and adhesion.^40^, ^41^ Increasing studies showed that TRIP6 was expressed in neurons of hippocampal and regulated biological function of neurological.^42^ Previous reference indicated that TRIP6 was sufficient and essential for proliferation and self‐renewal of NSCs, but suppressed NSCs differentiation.^43^ Another reference suggested that miR‐138‐5p modulated differentiation and proliferation of NSCs via inhibiting TRIP6 expression.^44^ In our reference, we exploited TargetScan software to find potential target gene of miR‐485‐3p and found that miR‐485‐3p has target sites in 3′‐UTR of TRIP6. Ectopic expression of miR‐485‐3p decreased the expression of TRIP6 in NSC. Dual‐luciferase reporter analysis was carried out to confirm that overexpression of miR‐485‐3p decreased the luciferase activity of pmirGLO‐TRIP6‐wt but not the pmirGLO‐TRIP6‐mut. Furthermore, we found that miR‐485‐3p reduced proliferation and induced differentiation of NSCs via targeting TRIP6 expression.

In summary, this reference revealed that miR‐485‐3p expression was down‐regulated during NSCs differentiation and miR‐485‐3p reduced proliferation and induced differentiation of NSCs via targeting TRIP6 expression. These data suggested that a crucial role of miR‐485‐3p in self‐proliferation and differentiation of NSCs. Thus, altering miR‐485‐3p and TRIP6 modulation may be one promising therapy for treating with neurodegenerative and neurogenesis diseases.

## CONFLICT OF INTEREST

The authors confirm that there are no conflicts of interest.

## AUTHOR CONTRIBUTION

Juxian Gu, Rusheng Shao, Meng Li, Qiuyue Yan, Hongwei Hu designed and conducted the experiments and analysed data. Juxian Gu wrote and revised the manuscript.
